# A Practical Investigation of the Therapeutic Value of the Salicylate of Mercury

**Published:** 1888-06

**Authors:** Henry T. Inge

**Affiliations:** Mobile, Ala.


VOL. V. JUNE, 1888. No. 4.

### Original Communications.

### A PRACTICAL INVESTIGATION OF THE THERAPEUTIC VALUE OF THE SALICYLATE OF MERCURY.*

BY HENRY T. INGE, M. D., MOBILE, ALA.

This new therapeutic agent has been known to the profession but a short time; consequently its value will be hard to determine, owing to the fact that it has not yet been generally manufactured. I was unable to procure any till September, when a New York druggist succeeded in buying an ounce from E. Merk, a druggist in Germany.

I have since then given it a fair trial in a few cases where mercury was indicated. The salicylate of mercury is a white amorphous powder, free from odor, tasteless, and slightly acid to test paper.

It is slightly soluble in water and alcohol. It contains .02 per cent. of water of crystallization and 59 per cent. of mercury.

*A paper read before the Medical Association of the State of Alabama.

Those who have written about this drug have limited their remarks to its use in syphilis.

Dr. Bruno Chaves, of Rio de Janeiro, in an article published in the *Medical and Surgical Reporter* of April, 1887, said that he had used it with marked results in many cases of syphilis, both internally and externally. I am inclined to differ with him, however, as I do not think the salicylate nearly so valuable in general treatment as the bichloride. I have given both a fair trial, and do not doubt the effects of bichloride are more prompt as well as more lasting.

My usual course is to begin mercurial treatment as soon as the secondary symptoms appear. To show the comparative value of the two preparations, I will give the history of two cases that came under my treatment at the same time, a man and his wife. I was treating him first. He had mucous patches and rubeola from head to foot. I gave him the following prescription—Rx. Salicyl. Hyd. grs. iv, Compound Tincture Gentian ℥1, Syr. Aurantii ℥ii Aqua ad. ℥vi M. sig. Take teaspoonful three times a day. His general health improved, but I could not see that the syphilitic lesion did.

I continued this treatment for twenty-seven days, and when the eruption disappeared, attributed it to the natural course of the disease, and not to the medicine. His wife came in, on the fourteenth day of his treatment, literally covered with pustules. I gave her the same prescription, substituting bichloride, 3 grs., for the four grains of salicylate of mercury. On the eighth day the pustules began to disappear, and ten days after there was not one on her body. I did not give up my treatment, but increased the dose of each, so that the man was taking five grains of the salicylate to the ℥6, and the woman four of bichloride. The latter continued to improve and had no other lesions, while the former showed symptoms of tertiary syphilis. I increased his dose to eight grains, but with no good effect. When I had about determined to change him to the bichloride, he began to improve and I did not see him for two weeks when he informed me he was well. I asked him if his bottle was empty. He replied that I did not want to cure him, so he had been taking his wife's medicine. These cases were under treatment in September and October, and, as is always the case with negroes, they discontinued treatment as soon as all external evidence of disease disappears. I saw the woman February 24th, when she miscarried with a four-months-old child, as was to be expected. I gave her the following prescription and will continue it for sometime if possible :

Rx. Hydrarg. Chloride cor. grs. iii, Potassi. Iodi. ʒvi, Tr. Gent. com. ℥i, syr aurantii ℥ii, Aqua ℥vi, M. sig. Take teaspoonful three times a day.

I have used the salicylate of mercury in twelve cases of syphilis, and in only two instances did I see any material improvement.

One was a young man with no external appearance of disease, but who had severe periosteal pains, especially in his feet. I gave him the twelfth part of a grain three times a day, and in three days he showed improvement that I couldn't get with the bichloride. The second case was that of severe headache after five years of inoculation. The pain disappeared after four days' use of the twelfth of a grain three times a day.

I have used this drug also in a few cases of non-syphilitic skin eruptions with some success. I had been treating a case of erythema circinatum for several weeks without any appreciable effect. I ordered an ointment of the strength of ℈ii to the ounce of lard, and the effect was remarkable. The patient was a farmer in good health; he stated that he had the eruption before, but that it would disappear until the present case which rendered him unable to work. This is a rare form of skin disease, and the only case I have ever had, and I think its cure with this agent worthy of remark, owing to the fact that I had previously used other forms of mercurial inunctions, chrysophanic acid ointment, oxide of zinc, and in fact everything I thought could possibly cure the trouble. I have used as a powder to apply to herpes so often found on the glans penis and prepuce the following: Rx. Hyd. Salicyl. grs. x, oxide of zinc ℈ii, bismuth sub. nit. ʒiss. Have never had any trouble with herpes, as this powder soon dries up the eruption. The salicylate of mercury has proven of undoubted value in eczema. The worst case of vascular eczema I ever saw proved amenable to its use.

Female prostitute, twenty years of age, has had eczematous eruption on left leg for three years with a deep and unhealthy ulcer on the external malleolus. Started treatment Feb. 12th with the following prescription : Rx. oxide of zinc ʒvii, Salicyl. Hyd. ʒi; used also adhesive strips above knee and covered entire leg with roller bandage. Dressed limb three times per week, using above powder freely. Discharged her well March 3d. Time will prove the ultimate result.

I had also a good cure in a case of eczema cruris in a negro woman, aged forty-five years. The disease was of three years standing, situated on the anterior part of the leg. She had been unable to walk for fifteen months, and had tried every remedy she had heard of to no purpose. I gave her this prescription to be applied twice a day after bathing the parts with warm water and castile soap:

Rx. Salicyl. Hydrarg. grs. xii liq., picis. ʒiii, cerate adipis ℥i. The effect was very marked, for in thirty days there was not an unhealthy spot on either leg, nor has the disease returned, although it was five months since that she was treated. I have tried this remedy, however, in a great many skin troubles where it had an appreciable effect. I have used it in diarrhœa of children, when the stools were of a greenish, muco-purulent character, with the best success, giving the fortieth part of a grain three times daily. My attention was directed to its use in such cases by the fact that I had used the bichloride with some success. The salicylate of mercury, combined with belladonna and other remedial agents, forms an excellent combination for applying it to the hands and feet when there is a superabundance of perspiration.

The much-lauded bichloride of mercury, as an antiseptic in the treatment of surgical cases, will, in time, give place to the salicylate. From its name we see that it combines two of the most valuable antiseptic agents. The mercury, which is a form of the acid nitrate, is precipitated with the salicylate of potassium and water, forming a new chemical agent different from either in decided chemical reaction, but preserving the antiseptic properties of both. In this, the day of progress, when all diseases are being investigated with a view of finding the germ, all remedial agents increase in value as their efficiency in destroying micro-organisms is proven. Soon their value will be solely for their ability to destroy germs. When that day comes, I shall claim a high place for the salicylate of mercury.

Throughout my report, you will notice that it had the best effect in disease caused by bacteria. The following quotation from the *Medical Record* shows that it must, in the nature of things, be a better antiseptic than the bichloride, as it states that an acid is essential to a perfect antiseptic:

"Dr. Ernest Laplace, of New Orleans, has, since last March, been working in Berlin under the direction of Dr. Koch. He has been engaged chiefly in bacteriological investigations, and one result of his labors will, no doubt, have great influence upon the future of surgical dressings. Bichloride of mercury, demonstrated by Dr. Koch in 1881 to be the best of antiseptics, did not do all the work that had been anticipated for it in surgery. It was found that as soon as a sublimate solution came in contact with an albuminous fluid, the albumen coagulated and formed with the bichloride a preciptate, which had lost all disinfectant power. Dr. Laplace proved by a series of experiments that not only is the precipitate devoid of disinfecting power, but also the supernatant liquid. In order to retain in dressing all the value of the sublimate, it may be absolutely necessary to prevent the formation of such a precipitate. After many fruitless experiments, he found at last the principle of the action which he was seeking, the addition of an acid to the solution of corrosive sublimate; such an acidulated solution will not form an insoluble albuminous precipitate. Dr. Laplace usually added five parts of hydrochloric acid or tartaric acid to a thousand parts of a solution of corrosive sublimate, though occasionally he substituted tartaric or carbolic acid. Of this acid solution Dr. Laplace says that, first, it makes no deposit of sublimate after standing, as all the sublimate solutions do, thereby retaining permanent strength ; second, when brought in contact with an albuminous fluid, the albumens will remain in solution and the whole strength of the solution of sublimate will be obtained as in the non-albuminous fluids; third, an acid medium is unfavorable to the development of micro-organisms. The addition of an acid to a sublimate solution also increases its disinfecting powers, so that a much weaker solution is required."

The dose given internally can range from the tenth of a grain up, as the case demands. The advantages of the salicylate over the bichloride may be summed up as follows:

1st. It is supported easily by the stomach without bad effects.

2d. It has decided curative effects on mucous patches and syphiloderms when used externally.

3d. It does not produce mercurial ptyalism.

4th. It is of great advantage in the treatment of a parasitic disease.

5th. It is effective when the bichloride has no effect.

